# The Impact of the Free Swimming Programme in a Local Community in the South East of England: Giving with One Hand, Taking Away with the Other

**DOI:** 10.3390/ijerph120404461

**Published:** 2015-04-22

**Authors:** Themis Kokolakakis, Athanasios Sakis Pappous, Steve Meadows

**Affiliations:** 1 Sport Industry Research Centre (SIRC), Sheffield Hallam University, Sheffield S10 2BP, UK; 2 School of Sport and Exercise Science, University of Kent, Chatham ME4 4AG, UK; E-Mails: A.Pappous@kent.ac.uk (A.S.P.); S.Meadows@kent.ac.uk (S.M.)

**Keywords:** health impact, swimming participation, Active People Survey, logistic analysis

## Abstract

The purpose of this study is to examine the impact of the introduction of the Free Swimming Programme (FSP) in a local community (not identified to preserve anonymity) in the South East of England. The question has been approached in a variety of ways: by using primary quantitative data from leisure centres and logistic regressions based on the Active People Survey (APS). Problems are identified related to the introduction of the FSP in this community and suggestions are made for future policy. A brief examination of swimming participation in England enables researchers to place this community into a national context. The problems and policies of sport organisation developed in this community are not dissimilar to a more general application reflecting the English experience; in this sense it is anticipated that the findings will enable managers of sport organisations, along with public health policy makers, to focus more effectively on raising sport participation. The unique selling points of this article are the examination of FSP for adult participants, the local analysis of junior and senior participation, and the overall assessment of the policy based on APS.

## 1. Introduction

The Free Swimming Programme (FSP) was announced in June 2008 as part of the UK Government’s Legacy Action Plan for the London Olympics in 2012, specifically focused on getting more adults physically active and providing young people with more physical education and sporting opportunities. It was funded by five Government departments: the Department of Culture, Media and Sport (DCMS), the Department of Health (DH), the Department of Education, the Department for Work and Pensions and the Department for Communities and Local Government. Financial and resource investment was also provided by Sport England (SE) and the Amateur Swimming Association; the latter co-ordinated and managed a team of County Swimming coordinators. The £140m funding for the FSP, initially over a two year period, was divided into four pots which promoted free swimming in two age groupings. These were revenue funding for those aged over 60 years (£15 million per year), those aged under 16 years (£25 million per year), capital funding for dissemination in the first year (£10 million) and further capital funding for dissemination, through a bidding process, over a two year period (£25 million per year). Additional funding was also provided by some local authorities to secure delivery of the FSP, but this replicated the same age groupings. Overall, 261 local authorities adopted FSP: 197 offered it to those under 16 years and over 60 years old; 64 offered it solely to over 60 years old [1]. There is a long tradition of focusing policy on those age groups on the grounds that they possess a lot of free time which is a vital element necessary for any increase in participation to actualise. For example, recent research [2] has found that the supply of swimming pools influences primarily the sports participation of the young and the over 65 age group. In addition there are significant health arguments related to obesity and functional capacity (introduced in the Literature Review). 

Both Central and Local Government justifiably regard swimming as a policy tool because it is a popular sport among a wide variety of age groups. According to our analysis of the Active People Survey, swimming has the highest latent demand with 5.4 million adults wanting to swim more often (12.9% of the English population). This fact was supported by a local sport participation survey conducted on behalf of the local authority in June 2009, which identified that 36% of respondents indicated that swimming would be the choice of activity they would most like to do, or do more often [3]. 

More evidence comes from the Welsh government’s implementation of a FSP for young people in 2003, at a cost of £2.4 million per annum. The Welsh programme's evaluation concluded that the maximum level of engagement occurred in the places with the greatest level of deprivation, indicating that, for these places, price was a barrier to entry [4].

In their “Evaluation of the impact of Free Swimming, June 2010”, commissioned by the funders of the FSP, PricewaterhouseCoopers LLP [1] reached the conclusion that under FSP more free swims were undertaken by young people under 16 years, than by people over 60 years old. On average, for each month of implementation, there were 23,000 and 114,100 additional swimmers over the age of 60, and under the age of 16 respectively, throughout England. Less evidence was presented on what actually happened in the 16–59 intermediate age group. Many children benefited from free swimming lessons, however demand soon outstripped supply to such an extent that lack of swimming pool capacity and waiting lists became important issues. The annual funding for the FSP for young people was £19.6 million [1]. In its scope, the aforementioned study did not deal with the impact of FSP on adults and the effect on local community swimming activities. The current research addresses indirectly the empirical findings of Gratton and Taylor [5] against the underlying assumption of FSP that price is the most important constraint to swimming participation. According to Gratton and Taylor [5] demand, in most sport facilities, is inelastic, *i.e.*, users (within limits) are not very sensitive to changes in prices; some are not even aware of price changes. Research on free entrance must be differentiated from any other research focusing on free attendance as they don’t share the same theoretical or practical background. Attendance, usually passive, is constrained from price/income and time spent, while participation includes, in addition, effort and involvement which limits the field of participants. This is a crucial difference in the appraisal of FSP. 

The aim of this research study was to evaluate the impact of FSP in a South East England community by analysing data collected by the local authority’s leisure management team from the four facilities that offered the FSP and by comparative analysis of the Active People Survey (2009–2010). Of particular interest is to what extent FSP can be associated with changes in the participation rates among target groups (less than 11 year old, less than 16 year old, over 60s) and among the population as a whole. The general effect of FSP on the population remains an issue relatively unexplored. This research argues that despite the intention to increase swimming participation, and thus improve health among the community, FSP created a series of unintended outcomes which impacted negatively on sports engagement. 

## 2. Literature Review

One function of the Choosing Health consultation [6] was to develop an activity plan for the UK population that would contribute to the delivery of “Game Plan”, the strategy for realising the Government’s sport and physical activity objectives. Game Plan set out a vision for increasing participation: to get 70% of the population performing 30 minutes of moderate intensity exercise five times a week by 2020 [7]. 

The 2012 London Olympics gave sport and health promotion a fresh impetus, and moved it higher up the Government’s public health agenda. It is recognised that sport and physical activity, along with a healthy diet, are key determinants of health [8]. Regular physical activity is associated with improved mental health and a reduced risk of cardiovascular and metabolic disease, obesity, osteoporosis and colon cancer [6]. The FSP specifically targeted children and older adults. Increasing physical activity levels in children would help to reduce the levels of childhood obesity, and diseases later in life [9]. In older adults, physical activity is associated with increased functional capacity [10]. Combined, these health benefits would help to secure improved health for the local and national community.

### 2.1. Main Theoretical Motivation

The main theoretical framework of this study is the Becker’s [11] model of labour and leisure choice, which assumes that agents derive satisfaction from consuming ‘basic’ commodities (such as going to the theatre, a meal or sports participation). The production and consumption of those commodities represents time out of work. Consumer choice models of sports participation indicate that agents have to decide if they participate at all and the amount of time spent in participation [12]. In this way, sports participation can occur directly by committing goods and time in the production or consumption of sport, or indirectly through acquiring social capital that eventually may lead to sports participation [13]. The latter is a critical parameter, usually underestimated in the vast majority of models aiming to explain sports participation. The indirect motivation of sport is usually actualised through active citizenship and cultural participation. In the last four years, it has acquired greater importance in the research agenda in the UK. The 2010 Mindshare Report for Sport England [14] investigated factors that correlate with swimming participation, across local authorities and based on the Active People Survey (APS). It was found that attendance at cultural events was a significant factor of sport participation “with those attending three or more cultural events swimming more frequently than those who attend fewer events” [14] (page 55). Finally, the Economic and Social Research Council, in its recent specification of the “What Works” programme in Culture Sport and Wellbeing required investigation into “the extent to which some culture or sports experiences might be mutually re-enforcing or in conflict or tension” [15]. The question is at the heart of evaluating free swimming participation, because, paradoxically, the answer from the aforementioned Sport England report is that a little cultural participation (less than twice per year) is in tension with sports participation, but much more cultural engagement is not. 

### 2.2. Ecological Argument

In deciding which factors are important in the analysis, both an ecological and an economic framework can be followed. The ecological perspective [16,17] acknowledges wider social and environmental influences on participation. An ecological approach does recognise intrapersonal factors (individual factors like demographics, biological, psychological and family situation), interpersonal factors (social support, partners for social activities, *etc.*), environmental factors (neighbourhoods, transport, weather, *etc.*) and societal and legislative factors (health sector policy, media and marketing, advocacy, *etc.*) as potential influences on behaviour.

Research has revealed various factors that can influence participation, which can be split into two broad groups—facilitators and constraints [18]. Facilitators are factors, either assumed by researchers or perceived by individuals, to enable or promote the formation of leisure preferences and encourage participation. Constraints on the other hand limit the formation of leisure preferences and inhibit participation in leisure [19]. This approach assumes an inherent motivation, implying that if individuals do not participate, it is due to constraining factors that prevent them [20]; this is the underlying philosophy of the FSP. Whilst it is important to acknowledge that entrance charges do present a constraint, the assumption is that the basic human condition is one involving an inherent desire to participate, and it only needs the constraints to be overcome to facilitate participation [18]—which is a simplistic understanding of a potentially complex decision making process. 

The composite price for leisure activities consists of both fixed and variable costs, including the cost of equipment/supplies, admissions charges, transportation, consumables (food and drink) and childcare costs [21,22]. An individual consumer will only invest in a good, or service, if the perceived benefits outweigh the costs [22]. This concept of cost/benefit weighing-up has been identified as a common feature in a number of other health behaviour models such as the Health Belief Model or the Theory of Planned Behaviour [17]. Gratton & Taylor [22] suggest that the decision to participate, or not participate in leisure activities, is most likely to be based on the total cost of the activity, whereas the frequency of participation is most likely to be based on the variable cost. Admission charges were found to be an important barrier to local residents participating in recreational physical activity [3], but not enough time, poor health/disability were more frequently cited. 

A study in Canada found that the impact of admission charges decreased with age and income, and affected both genders [23]. This observation was supported by the local council’s own physical activity survey that reported that younger age groups were more likely to give expense as a reason for not doing recreational physical activity, than older age groups [3]. In the same survey, poor health/disability was more likely to be cited as a reason for non-participation with increasing age—a third of those aged 60–64 and more than half (59%) of those aged 65+ cited these reasons. Interestingly, those with a disability were almost half as likely to cite “too expensive” as a reason not to participate. The findings from the local council’s own physical activity participation survey reiterate the importance of considering all the factors (ecological approach) that might constrain and/or facilitate participation in recreational leisure activities, rather than just the cost of admission [3]. They also reflect the rationalisation process individuals undertake to weigh-up the costs and benefits of participation/non-participation and the importance of considering the social circumstances (single parent, age, employment, *etc.*) and the competition they face for limited resources. Coalter [21] has argued that lowering entrance charges may not be an effective approach to increasing leisure participation, and other factors need to be taken into consideration. Taylor [24] observed that even though the prices charged for swimming increased above the rate of inflation in the late 1980s and early 1990s in the UK, this did not result in a significant reduction in swimming attendance, suggesting that attendance at local authority leisure centres is insensitive to substantial increases in admission charges. However, the impact on frequency of attendance at leisure centres may not be so resistant to price changes. Coalter [25] concluded that increased admission charges have little relevance as an absolute barrier to participation, but could influence frequency of use, which concurred with the work of Kay & Jackson (1991 cited in [25]).

### 2.3. Economic Argument

A different approach is provided by the economic perspective on sports participation emphasising the relevance of economic variables and the time related opportunity cost. 

The literature provides evidence that lower income may act as a barrier to sports participation [26,27,28,29,30,31]. Other studies have considered work-time’s negative association with sports participation [30,32]. Since time is finite, any rise in time devoted to sport will always be limited by competing demands, such as leisure and work. Time-related constraints have been considered as some of the most important barriers to sports participation [33]. 

The available literature points to a contradicting nature between income and sports participation. As income increases, work time rises and free time becomes more limited, giving simultaneously a positive and a negative effect on participation: a positive associated with more income and a negative associated with less free time [31]. 

The economic analysis of sport demand by Downward and Riordan [34], based on General Household data, focuses partially on swimming. In their results swimming participation rises if there are children in a family (especially at pre-school or school ages), if the adults are married, if the adult is female, or if there is car ownership. This contrasts to the general result from most research studies of increasing general sports participation among men (this is the case in all APSs). The same research emphasises the importance of social capital, an idea which is also underlined in Stempel [27] and is followed up in the present article. Breuer, Hallmann and Wicker [35] applied the socioeconomic model of sports participation on individual sports. In the case of swimming the two most influential variables, positively affecting participation, were human capital and gender (females). An extension of this model by Hallmann, Wicker, Breuer and Schonherr [2], integrates infrastructure into the basic socioeconomic model, showing substitution effects among activities. A poor supply of swimming pools was found to correlate negatively with the sport participation of young people (from 3 to 18 years) and the older age group (over 65 years).

Another article by Downward and Rasciute [36], examines the demand of sport as part of the demand for leisure as a whole, emphasising among others, the role of education in the process. They found that there is a substitution effect between sport demand and other leisure opportunities, which again raises many more questions in terms of how this interdependence is formed and influenced. In a parallel way Wicker, Hallmann and Breuer [37] examined, using multi-level analysis, micro socio-demographic factors of sports participation as well as macro factors such as swimming infrastructure. Downward, Lopez and Rasciute [13] applied a zero inflated ordered probit model on Spanish participation data, showing that the factors that affect the decision to participate or not are different than the factors that affect the frequency of sports participation. 

Finally, removing price to attract new participants and encourage existing ones can be linked to the widely used Ansoff matrix [38]: as the initial offer was simply swimming, no product development and diversification were possible; participants were either existing (market penetration) or new users (market development).

### 2.4. Conclusions

In conclusion, both economic and ecological frameworks point towards a model where sports participation is correlated with four key factors: health, culture, income/price, and free time; directions compatible with the initial theoretical motivation of Becker’s [11] work. All other variables considered come from this basic framework. Gender relates to cultural issues and income inequalities, age relates to health and free time [3], occupation relates to income and culture, education relates to culture and income, unemployment relates to both income and free time. The latter brings into play other variables such as retirement, and being a student. The cultural dimension assumed in this research is not incompatible with the Becker [11] model. For a given level of health, income and free time, people still require a cultural background to differentiate between sports participation and other leisure pursuits [36]. This conclusion is compatible with the results of the Academic Literature Review of Breuer, Hallman, Wicker and Feiler [39]. All variables examined in this research come out of this four key-factor framework. 

## 3. Methodology

FSP was introduced on 1 January 2009 in four local authority-run leisure facilities in an urbanised region of South East England for the aforementioned age groups. The region has five other sport/recreation centres under local authority control, one of these run by a private leisure service provider working in partnership. The activities on offer range from traditional sports centre activities through to golf, athletics track, gyms and body conditioning activities. Despite free entry, FSP resulted in simultaneously decreasing sports participation and increasing market penetration where the majority of participants were swimmers prior to its implementation [40]. Due both to government cutbacks, announced in 2010, and the FSP’s insufficient impact, the FSP funding was withdrawn in July 2010. In April 2011, with the financial support of the local authority, FSP was re-introduced for the over 60s and children under 11 years old. Free swimming was still available for local residents in 2012, but only for those in these two age bands. Of the four leisure sites with swimming pools, three had standard 25 m pools and one had a leisure pool.

The objective of the FSP was to get more adults and children active. It is widely recognised that sport can affect social change through health improvements, more inclusive communities, less crime, education and active leisure/citizenship [41]. The population of this area in 2009 was approximately 255,000 residents. When the FSP was commissioned, this community’s health profile indicated that 80.2% of 5–16 years old spent at least 2 h a week on high quality PE and school sport, which was significantly worse compared to the England average of 90% [42]. However, the number of obese children was significantly better than the England average [42]. The number of physically active adults aged 16+ years was reported as 5.7%, significantly worse than the England average of 10.8%, but the number of obese adults (25.3%) was not significantly different from the England average (23.6%). 4.6% of the population was diagnosed with obesity, which was significantly worse than the England average (4.1%). Life expectancy of males and females were both significantly worse than the England average, reflected in significantly worse deaths from smoking along with early death from cancer, heart disease and stroke [42]. The Department for Communities and Local Government’s *Index of Deprivation 2010* ranked the local authority’s eight most deprived areas, within the 10% of most deprived nationally. This statistic has grown noticeably between 2007, when there were five deprived areas in the bottom 10%, and 2004 when there was only one. In the 10–11 year old age group, 20% are classified as obese, while estimated levels of adult healthy eating and obesity are worse than the England average [43]. This picture suggests that this local community had an urgent need to raise the physical activity levels and health of its residents. This evidence makes the focus on sport participation much more urgent. Despite efforts to raise public awareness towards the risks of a sedentary life, an increasing number of elderly and adolescents are showing low levels of physical activity [44].

There is no straightforward comparison between the English population health average and the health of people in this local community in South-East England. Deprivation is lower than average, however there are children who live in poverty and the life expectancy for men and women is lower than the English average [43]. Despite the local character of this research, some trend results (although not the entire analysis) can be generalised across the UK, as health related issues, such as obesity and the stagnation of sports participation, are a general recurrent theme amongst most local authorities. 

### 3.1. Data Collection and Analysis

*Quantitative data* was collected at source in four leisure centres. The information was collected via cash registers into a LeisureFLEX computerised system; every time a swimming transaction was made it was recorded on the system. A swimming report was produced by extracting data from LeisureFLEX database using Cascade3D software. This data extraction was carried out by the Leisure Services Manager for the local Council.

In this paper the Active People Survey (APS) was also used to explain the pattern of adult swimming participation. Unfortunately, this does not extend to junior participation, which must be analysed on the basis of the primary research. 

Before analysing the local community’s participation, we provide a useful contextual framework of the issue by considering swimming participation in England as a whole, and testing the overall significance of the FSP in England. In this sense the three stages of our analysis are:

- Analysis of swimming participation in England through APS
- Analysis of swimming participation in the local community through APS
- Analysis of swimming participation in the local community through primary research data collection

The dataset chosen for this analysis was APS4 (2009–2010). This coincides with the first year of the FSP, containing measurement parameters that address the uptake and awareness of the programme. Hence when FSP takes the value 1, the associated area has an active FSP; while zero indicates non-active FSP. The APS survey was conducted through a telephone questionnaire. The valid sample size used in this analysis is 185,591. Although, FSP-related variables are important in modelling sports participation in the general English case, they offer no benefit (within APS4) in the case of this local authority, as all its swimming pool facilities participated in FSP. The logistic regression technique is used to model swimming participation. The regression model is estimated using Binary Logistic Regression in SPSS. All the explanatory variables of the model are binary capturing the factors: Gender, Age, Education, Occupation, FSP and Culture, as discussed in the Literature Review. An important statistic associated with each variable is the odds ratio, defined as the ratio of success (*p*) over failure to participate (1 − *p*) associated with each variable *X*:

Odds ratio = *p*/(1 − *p*) = e*^βX^* (1)

The advantage in using a logistic non-linear model rather than ordinary least squares (OLS) is that the expected participation rates generated from the former are designed to have a minimum value of zero and a maximum of one. This makes it ideal for binary variables such as participation (0 stands for non-participation and 1 for participation). The results are presented and analysed in terms of the estimated coefficient values (B), or their odd ratios. Of vital importance is the selection of the reference variable. For example in Table 1, gender was reported as male (1), implying that female (0) is the gender reference. A change in the reference category will bring about different coefficients without changing the logical interpretation of the result. To explain the concepts, a negative coefficient in the gender variable (−0.53) implies that as we “switch” from female to male, the log of the odds ratio declines by 0.53. This is not easy to interpret. For this reason the odds ratios in Table 1 are used. In the case of gender the odds ratio is e^−0.53^, equal to 0.59. It implies that as we switch from female to male, the odds ratio to participate changes by a factor of 0.59 (a decline of 41%). Obviously, the odds ratio can only take positive values.

### 3.2. Econometric Modelling

The starting point is to construct a logistic model for England as a whole, using the APS4 dataset, which corresponds to the first year of the implementation of FSP, 2009–2010. Our dependent variable is swimming participation, meaning participating at least once for at least 30 minutes (and at least moderate intensity) in four weeks. Independent variables include gender: male (hence using female as a reference); occupation: professional, skilled non-manual, skilled manual, partly skilled, long term unemployed, full-time student, (reference: managerial occupation); Age: 25–34, 35–44, 45–54, 55–64, 65–74, 75+, (reference: 16–24); education: no qualifications, lower and medium education (reference: higher education). These factors correspond to the APS categories, and also reflect the analysis presented in the Literature Review in terms of income/price, free time, culture and health. To avoid the problem of multicollinearity, the independent variables included in the model had an absolute correlation of less than 0.7, which is below the suggested cut-off criteria of 0.9 [45]. The FSP variation is captured in APS4 where the level of swim offer by local authority is indicated as:

- 16 and under and over 60
- 60 and over only and
- not offered

Finally, following the argument in the literature review, two variables on cultural participation were included: “attending creative arts last year” and “doing creative arts last year”. An important insight is provided by the Taking Part Survey [46], commissioned by DCMS and its partner Non-Departmental Public Bodies (NDPBs), such as Sport England and English Heritage. According to the Taking Part Survey [46], cultural participation (and participation in general) tends to be higher among upper socioeconomic groups even in the least deprived areas of England. This is also a pattern observed in the case of sports participation in general. As Table 1 suggests, even when different socio-economic positions were accounted for, there is a strong significance in the logistic regression framework between sports participation and cultural participation, indicating that the dynamics of the relationship goes deeper than a simple financial statement of income or employment position. The common ground is social capital participation and citizen association in general which, as demonstrated in the Taking Part Survey, is following this pattern. This is a new concept in participation modelling and it is the logical outcome of the article by Downward and Rascuite [36] showing a substitution effect between sport demand and leisure opportunities. It also underpins the sport-related ESRC funded research on Wellbeing in the UK. People that become more active in the civic sense are likely to choose more active forms of entertainment such as sports participation or “doing” creative arts, at the expense of more passive forms of entertainment such as watching TV. Finally note that the inclusion of a variable able to capture a form of social capital (such as cultural participation and education) is validated by Becker [11] and subsequent research [13]; sports participation may be generated through a “deposit” of social capital, while the latter is not necessarily acquired for the purpose of sport. In most models (see the economic argument in Literature Review), this is accounted solely by education, which is the reason for educational qualifications appearing to be, in much applied research, so strongly significant. 

## 4. Results and Discussion

### 4.1. Quantitative Analysis for England

The resultant model for swimming participation in England can be seen in Table 1. 

**Table 1:** Swimming sports participation in England—English model (APS4).

Variables	B	SE	Odds Ratios	*p*
Constant	−1.30	0.35	0.270	<0.01
FSP juniors and seniors (*reference: no FSP*)	−0.072	0.019	0.930	<0.01
*FSP seniors only (reference: no FSP)*	*0.15*	*0.023*	*1.016*	*0.505*
Gender: male (*reference: female*)	−0.529	0.015	0.589	<0.01
Age: 25–34 (*reference: 16*–*24*)	−0.066	0.028	0.936	0.017
Age: 35–44 * (*reference: 16*–*24*)	0.049	0.028	1.05	0.078
Age: 45–54 (*reference: 16*–*24*)	−0.210	0.028	0.811	<0.01
Age: 55–64 (*reference: 16*–*24*)	−0.381	0.032	0.683	<0.01
Age: 65–74 (*reference: 16*–*24*)	−0.634	0.046	0.530	<0.01
Age: 75+ (*reference: 16*–*24*)	−1.55	0.057	0.213	<0.01
Education: medium (*reference: higher*)	−0.122	0.019	0.885	<0.01
Education: lower (*reference: higher*)	−0.300	0.021	0.741	<0.01
Education: no qualification (*reference: higher*)	−0.525	0.03	0.591	<0.01
*Occupation: professional (reference: managerial)*	*0.006*	*0.033*	*1.006*	*0.845*
Occupation: skilled non-manual (*reference: managerial*)	−0.172	0.021	0.842	<0.01
Occupation: skilled manual (*reference: managerial*)	−0.185	0.023	0.831	<0.01
Occupation: Partly Skilled/Unskilled (*reference: managerial*)	−0.269	0.025	0.764	<0.01
Occupation: unemployed (*reference: managerial*)	−0.572	0.045	0.564	<0.01
Occupation: student (*reference: managerial*)	−0.150	0.034	0.861	<0.01
Retired (*reference: opposite*)	0.094	0.034	1.098	<0.01
Attending creative arts last year (*reference: not attending*)	0.351	0.016	1.420	<0.01
Doing creative arts last year (*reference: opposite*)	0.078	0.016	1.081	<0.01

N = 181,652. Insignificant variables: “Occupation: Professional”, “FSP seniors only”; * Significant at the 10% level.

The effect of FSP on the adult population is strongly significant and negative. It implies that the FSP in the first year of implementation had a negative impact on adult participation across England. Following the introduction of the FSP, the odds ratio to participate in swimming among adults declines by a factor of 0.93 (*i.e.*, 7%). This immediately raises questions about the wisdom of Government intervention and the impact of free swimming entry on existing users (Table 2, later on, shows a cross-tabulation of the participation rates against FSP, illustrating further this argument). Some of the arguments mentioned (e.g., swimming participation in general) are not specific for the intervention; however they are necessary for establishing a contextual base for the discussion. 

Sport participation tends to be higher in men than women [31], a pattern that emerges for each year of the APS. However, in the case of swimming, the variable male (Table 1) has a negative coefficient, implying that compared to women, males are less likely to be swimming participants. Following a change from a female (reference) to a male, the odds-ratio to participate changes by a factor of 0.59, *i.e.*, corresponding to a decrease of 41%. 

**Table 2:** Swimming participation: at least one 30’ session in four weeks, adults, APS4/3.

APS	FSP	No FSP
Swimming participation APS4	12.9%	13.3%
Swimming participation APS3	13.2%	13.3%

Analysis of APS3 and APS4 using local authority weights.

All age categories above 16–24 years (reference), apart from the 35–44 years group, have negative coefficients. The latter is the most important age category in terms of swimming participation. Its odds ratio (1.05) implies that when the age range is switched from 16–24 year olds to 35–44 year olds, the odds to participate increase by 5%. Outside this, the remaining age categories follow the expected pattern, with coefficients declining as age increases, implying a reduced probability to participate among older groups. 

The educational factor behaves exactly as expected from aforementioned sport participation studies. A higher level of education correlates with higher swimming participation. All educational variables in the model in comparison with the reference category of higher education have negative coefficients, illustrating that as we switch from higher education to lower or no qualifications, there is a decline in the odds ratio to participate. Further, as the odds ratios illustrate, the (positive) effect on participation is stronger when the education standards improve. For example, as higher education is substituted for no qualifications or medium education, the odds ratio to participate declines by 41% and 12% respectively; the most qualified group declines the least. After examining the APS dataset it was decided to include occupation rather than income, as the latter had too many unanswered questions that could have deteriorated the strength of the findings. In the occupational categories, the managerial reference (together with the professionals who are not significantly different to the managerial category) is the occupational group most likely to provide swimming participants. All other categories have negative signs. Among the working groups, the decline of the coefficients and the odds ratios are analogous to the occupational hierarchy. The full time student group is the third most prominent group (according to its odds ratio). The long term unemployed are the most disadvantaged, in terms of swimming participation, having the most negative coefficient. As the reference category: managerial “switches” to unemployed, the odds ratio changes by a factor of 0.56 (*i.e.*, a decline of 44%). Retired people have a positive coefficient, implying that the point of retirement is a positive factor associated with swimming participation. Presumably this rise in participation is related to the increase in free-time available. As people become retired their odds ratio to participate increase by 10%. Finally, the two cultural variables are very significant and both correlate positively to swimming participation, implying that there is a strong link between cultural/citizen participation and swimming participation. In other words swimming and cultural participation appear to complement each other. For example, in the case of “attending creative arts”, the odds ratio is 1.42, implying that as this civic activity is introduced, the odds to participate in swimming increase by 42%; an equivalent increase was observed when introducing “doing creative arts” (8%). This has strong implications in terms of policy: addressing price alone is unlikely to deliver the desired participation increase, a fact verified by Gratton and Taylor [5]. The analysis above indicates that, among adults, swimming participation is driven by females, aged 35–44, having higher education, occupied in managerial or professional jobs, and being active in the civic sense (in this case attending or doing creative arts). From the outset, any local authority policy relating to swimming should at least address the issue of the effects on this group. As it stands, the FSP targeting increased accessibility to swimming for juniors and seniors but had an overall negative effect on the adult population participation pattern for England as a whole. To illustrate this result Table 2 presents a cross-tabulation between swimming participation (at least one 30’ session in four weeks) and FSP programme for the years 2009, when FSP was active and 2008 when it wasn’t. In the case of 2008 we considered the same distribution of local authorities as in 2009 for the results to be comparable.

Stating from APS3 (2008), there is no significant difference in the participation rates between the local authorities that in 2009 enforced FSPs (13.2%) and those that did not (13.3%). Then, following the first year of FSP, the first group experienced declining swimming participation rates among the adults from 13.2% to 12.9% whilst the participation rate in the second group remained the same. Finally, as highlighted earlier, the introduction of FSP implies that the odds ratio to participate in swimming among adults declined by a factor of 0.93 (*i.e.*, 7%).

### 4.2. Quantitative Analysis for the Local Community

Having established the English swimming pattern and the groups that drive participation, the focus now shifts to the local community. Table 3 presents the results of the previous methodology applied on the local authority. The big difference now is a dramatic reduction in the sample size to just over 900. This means that the detail captured in the English sample is unlikely to be reproduced. However, the sample is still big enough to capture the most important relationships. As all the leisure centres participated in the FSP, there is no point in including anything FSP related within the set of independent variables; however a comparison with the general English case can provide useful insight. 

Under the APS4 dataset, in the Local Authority the gender and occupational variables do not appear as significant in affecting swimming participation. In terms of occupation, the general negative effect associated with partly skilled occupations (compared to managerial) is maintained although some categories such as skilled manual and unemployed are not significant any more. Full-time students fare worse than in the general English case. As occupation is switched from managerial to 16+ full-time students or partly skilled, the odds ratio to participate declines by 61% and 63% respectively. However, there are three significant reversals compared to the English results. The retired and active culture groups have negative coefficients, while the lower educational impact (compared to higher) has become positive. Retirement is only significant at the 10% level, but it suggests that the FSP policy may not have a positive effect at the point of retirement. In the case of “doing” creative arts, in the year of application of FSP, the sign of the effect in the community is the opposite of the English overall effect: people with high civic involvement stayed away from swimming. As the “doing arts” category is switched on, the odds ratio to participate declines by 50%. In other words, people with higher education, culturally active and even the retired are less likely to participate. This result was observed during the year that FSP was fully active in all the leisure centres, which raises the question of a strong relationship with the aforementioned policy. 

**Table 3:** Model for swimming sports participation in the local authority (APS4).

Variables	APS4			
	B	SE	Odds Ratios	*p*
Constant	−1.09	0.48	0.34	0.023
Education: lower (reference: higher) *	0.651	0.344	1.917	0.059
*No qualification (reference: higher)*	−*0.027*	*0.454*	*0.974*	*0.953*
*Education: medium (reference: higher)*	*0.336*	*0.323*	*1.400*	*0.298*
*Occupation: professional (reference: managerial)*	−*0.569*	*0.652*	*0.566*	*0.383*
Occupation: skilled non-manual (*reference: managerial*) *	−0.555	0.321	0.574	0.084
Occupation: skilled manual (reference: managerial) *	−*0.210*	*0.300*	*0.810*	*0.483*
Occupation: partly skilled (*reference: managerial)*	−0.981	0.392	0.375	0.012
Occupation: student *	−0.939	0.525	0.391	0.074
Retired *	−0.876	.500	0.416	0.080
*Long term unemployed*	−*0.912*	*0.6200*	*0.402*	*0.141*
Doing creative arts last year	−0.698	0.288	0.498	0.015
*Attending creative arts last year*	*0.146*	*0.217*	*1.157*	*0.501*

N = 904; * significant at the 10% level.

This implies two things: firstly, that the resource of swimming, in relative terms, became more limited; and secondly, in combination with the negative coefficient of “doing creative arts” that the leading group driving swimming participation stayed away. The effect of introducing FSP on the adult population in this local authority area was that the most active adult participants stayed away from the swimming pools. Starting from the position that an important barrier to participation is cultural, a policy of free entry should be seen as one that can increase attendance in the target group but not participation (in the sense of swimming for 30 minutes at moderate intensity). 

### 4.3. The Local Authority Leisure Centres Dataset

As mentioned before, local authority leisure centres started implementing the FSP from the 1st of January 2009, until July 2010, when it was interrupted by funding cuts. A modified version of the original policy (seniors and children under 11 years old) had been in place since April 2011. 

The local authority datasets provide headcount entries for swimming admissions, from January 2008 to September 2011. An example of the collected data is presented in Table 4, where there is a dataset from one of the facilities, together with our interpretation of the results into four age categories: Under-11s, juniors, adults and seniors. Entries into the swimming pool are also classified according to the nature of entrance into 17 categories (such as casual swim, concession cards, family ticket, schools *etc.*) as it appears in the first column of Table 4. Although some categories, such as Family, are ambiguous in terms of age distribution, the most important categories such as Casual Swim Adult, Casual Swim Junior, and Casual Swim Senior have a straightforward interpretation. 

**Table 4:** Local authority data: swimming pool dataset and classification.

January February and March 2009, Headcount				
Swimming Group	Headcount	Headcount	Headcount	TitlAge group
Casual Swim Adult	3109	3652	3867	Adult
Casual Swim Disabled	25	23	35	Adult
Casual Swim Junior	6375	7396	5210	Junior
Casual Swim Senior	569	723	881	Senior
Casual Swim under 3	381	510	607	Under 11
Casual Swim under 5	353	404	438	Under 11
Concession Cards	21	13	16	Under 11
Family Ticket	8			Junior
Fins Club Member	307	195	351	Junior
Free Ticket	2	3	2	Senior
Group Swims	2	1	1	Under 11
Miscellaneous	4	26	5	Under 11
Passport to Sport	284	165	234	Under 11
Schools	73	50	65	Under 11
Season Entry	100	45	141	Adult
Season Tickets	2	4	2	Adult
Swim Lesson Concessions	83	53	105	Under 11
Total	11,698	13,263	11,960	

The summary of results are presented in Table 5 as monthly average entries for each age category over five distinctive periods: 2008 (no FSP), 2009 (full FSP), 2010 (January–July, full FSP), 2010 August–2011 March (no FSP), 2011 (April–September, amended FSP). Participation figures have a strong seasonal element with very low figures in December/January and very high in the summer months. Hence, in order to account for both seasonality and different time periods, the figures are presented as average monthly admissions. Where required the figures were further seasonally adjusted; for example, monthly averages between the second (2009) and the third period (up to July 2010) cannot be meaningfully compared as the latter does not include the December period (worse month for swimming participation). 

Finally, on the basis of the seasonally adjusted statistics, a percentage change table is constructed from one period to another as shown in the last part of Table 5. Little changes in the figures cannot be attributed, with confidence, to FSP. However, in great swings an FSP influence can be established. By considering the 2009 changes, it is safe to say that the main impact of FSP on local resident’s participation was felt in the Under 11 category, the juniors and the seniors. The changes in the adult rates are not great enough to enable us to suggest such a link. Positive changes exist in juniors (57.8%) and seniors (109%). There are negative changes noted in the Under 11 participation which declined by more than 30%. The abolition of FSP in the fourth period August 2010–March 2011 reversed the 2009 effects. Participation among the Under 11 children increased on average by 83%, while participation among juniors and seniors declined by 50% and 42% accordingly. 

The re-introduction of the amended FSP policy in the final period saw dramatic changes in the under 11 year olds and the seniors categories increasing by 175% and 22% respectively. At the same time, as the last part of Table 5 indicates, the juniors’ entries (11–16) declined by 60% reaching a worse position than in 2008 when the original form of FSP was not in place. It is apparent that the younger than 16 year old group should not be treated as if it was a homogenous entity: it includes the 11–16 group which may enter a swimming pool without parental accompaniment and the younger ages which are linked to other adult participants. In effect the target group that benefited from the initial FSP was the 11–16 age group, while there were negative effects on the younger categories. 

This analysis verifies the original thesis from the analysis of the APS. A free entry policy increased the junior entries (without reaching the intensity of participation standards) at the expense of existing participants, including females and younger children. In business terms this is hard to defend as the latter groups are the long term regular customers of the swimming pools. 

**Table 5:** Local authority data (monthly averages), four leisure centres.

Time Period	FSP	Monthly Averages				Total
		under 11	11–16	Adults	Seniors	
2008	no FSP	2204	7999	8101	1205	19,508
2009	full FSP	1535	12,623	9068	2522	25,748
2010 (January–July)	full FSP	1695	10,680	9663	2397	24,436
August 2010–March 2011	no FSP	2509	4655	6978	1326	15,468
2011 (April–September)	amended	7800	2970	10,257	2270	23,297
		**Monthly Averages, Seasonally Adjusted**				
2008	no FSP	2204	7999	8101	1205	19,508
2009	full FSP	1535	12,623	9068	2522	25,748
2010 (January–July)	full FSP	1554	10,136	9057	2424	23,178
August 2010–March 2011	no FSP	2839	5086	7703	1414	17,212
2011 (April–September)	amended	7800	2027	8199	1719	18,901
		**Monthly Averages, % Changes**				
2008	no FSP					
2009	full FSP	−30.3	57.8	11.9	109.3	32.0
2010 (January–July)	full FSP	1.2	−19.7	−0.1	−3.9	−10.0
August 2010–March 2011	no FSP	82.7	−49.8	−14.9	−41.7	−25.7
2011 (April–September)	amended	174.8	−60.1	6.4	21.6	9.8

## 5. Conclusions

This research answers the main questions about characteristics of swimming participation and the impact of FSP in the local authority region. To establish a background, it is shown that increases in swimming participation in England are associated with younger age categories (peaking in the 35–44 years old group), wealthier and better educated people, those retired from work, and women. Cultural/citizenship participation is also established as a major factor with a positive correlation to swimming participation. However, within this context, in England overall, the first year of FSP appears as a negative influence to swimming participation among adults (based on APS). 

In the local authority the first year of FSP helped to increase the number of juniors (11–16 years old) entering the swimming pools. However the original unstructured approach did not translate necessarily into an equivalent (to entries) increase of their participation (defined as swimming for 30 min). The big lesson here was that the abolition of the price constraint did increase admissions but did not ensure actual participation. In this case adult swimming participants and their accompanied children stayed away deterred from a crowding out effect. By examining the participation data of APS in 2009 it was established that the application of FSP changed significantly the swimming environment and the leading group of participants. Within the FSP context, lower (rather than higher) education and cultural involvement are significant drivers of participation. 

In the second phase of FSP, with the focus on young children and seniors, a positive effect on swimming participation was observed among children and accompanying adults and a negative effect on junior entries 11–16 years old (compared to the high point of the first year). This research backs the current, more structural direction, of the local authority sport policy, aiming to increase long term swimming participation rather than just entries. It shows that participation in active recreation can increase through appropriate programme planning, aiming to reduce constraints faced by non-participants within a community. FSP alone cannot address this dimension. It also does not consider the financial viability of such an endeavour as in the long term; given the current economic direction, swimming pool facilities have to establish a degree of financial independence. The reality is that not every individual faces the same constraints, and these will vary depending upon intra-individual, inter-individual, and social-cultural factors. Hence, in designing such programmes leisure and recreation planners need to investigate non-participants and the constraints they encounter, and clearly differentiate amongst the different aspects of non-participation (such as in male swimmers and non-participants in this local authority). Clearly it is critical to appreciate the constraints and barriers individuals encounter in active recreation, but equally to ensure that there is awareness around the key factors that would facilitate participation. This avoids the situation of assuming participation would be automatic once perceived constraints are removed. Consideration should be given to the level of educational attainment and the level of engagement in community cultural activities. 

Many local authorities and leisure providers strive to create equal opportunities in terms of access to active leisure and recreation. This has resulted in them offering many services and programmes to communities on an undifferentiated basis. But the reality of the situation is that equity in provision of active recreation services requires the identification of clear target groups of non-participants and the specific barriers and constraints they face [20], along with a clear understanding of the factors that would facilitate engagement and expansion of their active recreation pursuits. 

In a comprehensive way, the challenge to improve health through increases in sports participation cannot be negotiated simply by removing cost barriers. This has applications throughout the health policy spectrum. For example the free gym sessions prescribed through the National Health Service for obesity, are not going to be actualised simply on the basis of being free. In planning such initiatives care should be taken to:

- Identify leading groups in the specific participation field and the impact of policy on them.
- Identify the non-financial/ cultural constraints of non-participants.
- Design initiatives that promote specific impacts rather than population-wide policies.
- Develop structured sessions for the participants (through prescription or specific programme).
- Emphasise the importance of a safe environment and support for personal development.
- Develop complementary features to maximise impact, such as continuous monitoring and high quality leadership.

This paper has provided a comparative analysis of the state of swimming participation in England (based on Active People Survey), along with the impact of FSP in a South East England local authority (based on dedicated database). The unique contribution of this research lies firstly in the identification of civic/cultural participation as a major determinant of swimming participation, and in showing that a dataset such as APS can be used at the local level to evaluate and inform policy rather than just analysing the past. The findings are consistent with analysis from Gratton and Taylor [5,22] and Downward and Rasciute [36]. Secondly, this analysis showed that any planned initiative needs to consider the potential impact on existing users/customers, so these are not lost. Finally, this research by differentiating the juniors into two groups: 11–16 and younger illustrated the opposite dynamics of engagement at play. Useful further research would be to interview local leisure managers on their experience of the introduction of FSP in the community as they can provide some feedback on useful lessons learned. Equally, interviews with users and non-users may provide an added dimension. This may lead to a more strategic implementation of similar future free schemes, as suggested by Coalter [47]. 
